# The application effect of the 5E-microteaching integration model in the standardized training of general practitioners

**DOI:** 10.3389/fmed.2026.1812102

**Published:** 2026-05-04

**Authors:** Linjuan Li, Yuxin Guo, Haimei Du, Ning Shi, Xiaorong Gao

**Affiliations:** 1 Department of General Medicine, Yan'an University Affiliated Hospital, Yan'an, Shaanxi, China; 2 Department of Endocrinology, Yan'an University Affiliated Hospital, Yan'an, Shaanxi, China; 3 Department of Neurology, Yan'an University Affiliated Hospital, Yan'an, Shaanxi, China

**Keywords:** 5e teaching model, general practice, microteaching, standardized residency training, teaching effect

## Abstract

**Background:**

This study evaluated the effectiveness of a training model combining 5E teaching and microteaching in improving clinical abilities, critical thinking, and learning satisfaction of general practitioners (GPs) in standardized training.

**Methods:**

Sixty general practice residents undergoing standardized training at Yan’an University Affiliated Hospital (Sept 2023-Sept 2025) were enrolled to minimize sampling bias, randomly divided into two groups. The experimental group received 5E microteaching (participation, exploration, explanation, expansion, evaluation) with microteaching for skill documentation and feedback; the control group received traditional teaching. Group differences were assessed using the MINI-CEX, CTDI-CV, graduation evaluations, and a satisfaction questionnaire.

**Results:**

No significant baseline differences (sex, age) validated randomization. The experimental group outperformed the control in all MINI-CEX dimensions (all *p* < 0.05), showed significant CTDI-CV improvements in truth-seeking, openness, analytical capability, systematicity, self-confidence, curiosity (all *p* < 0.05; cognitive maturity unchanged), higher scores in theory, medical record writing, operational skills (all *p* < 0.001), and higher satisfaction (96.67% vs. 73.33%, *p* = 0.030).

**Conclusion:**

The “5E-Microteaching (integrated model)” effectively improves clinical competencies, critical thinking, and satisfaction among general practice residents, providing an empirical framework for competency-based innovation in general medical education.

## 1 Introduction

General practitioners (GPs) serve as the primary “gatekeepers” of healthcare systems, a role that has become increasingly critical in China amidst its rapidly aging population and the escalating burden of chronic diseases (1). Recognizing this, the Chinese government has, since 2011, systematically established a nationwide standardized residency training (SRT) program for GPs, anchored by a “5 + 3” model (5 years of undergraduate medical education plus 3 years of specialized residency) to cultivate competent, compound healthcare professionals for primary care (2). This initiative, fully implemented in 2014, has now evolved into a mature, large-scale system: by the end of 2018, Shenzhen had enrolled 1,183 general practitioners in standardized training (3). However, despite its scale and strategic importance in advancing a hierarchical medical system and implementing the “Healthy China” strategy, the predominant teaching approach in these programs remains lecture-based learning (LBL) (4). LBL prioritizes systematic knowledge transmission but often places residents in a passive role, leading to documented deficiencies in clinical reasoning, practical skills, and complex case analysis (5, 6). Such a model falls short of meeting the demands of modern competency-based education, which emphasizes the cultivation of clinical acumen and autonomous problem-solving.

To address these shortcomings, there is an increasing emphasis on structured curriculum reform within medical education. Recent evidence underscores the pivotal role of curriculum integration and innovation in developing competency-oriented educational designs that meet contemporary healthcare demands (7). In this context, educators are exploring innovative pedagogies. The 5E Teaching Model, grounded in constructivist learning theory and originating from Robert Karplus’s 1960s inquiry learning framework, was formally developed in 1989 by Bybee et al. at the U.S. Biological Sciences Curriculum Study (8). This model restructures the learning cycle into five interconnected stages: engagement, exploration, explanation, elaboration, and evaluation. While traditionally prominent in science education for fostering active learning and knowledge integration, it has recently demonstrated promising applications in medical education, particularly in enhancing clinical decision-making through a problem-based structure (9, 10). Microteaching has become a classic method of teacher skills training since it was first proposed at Stanford University in the 1960s (11). This teaching method in medical education primarily relies on video practice and accurate feedback, which has been proven to significantly improve clinical operations and communication skills and offers the advantages of high standardization and proficiency (12). While valuable individually, each has inherent limitations when applied in isolation. The 5E model primarily cultivates cognitive and comprehension skills but may lack a focus on technical precision. Conversely, microteaching alone can lead to repetitive skill drills without embedding them in deep clinical reasoning. In the context of GP training—where practitioners must navigate complex, ambiguous presentations and perform a wide array of procedures—the ultimate goal is the seamless integration of cognition (clinical reasoning) and behavior (technical execution). To date, no established pedagogical model has systematically integrated these two complementary approaches to address this cognitive-behavioral gap.

To bridge this gap, we developed and evaluated an integrated “5E-Microteaching” model that combines the cognitive scaffolding of the 5E framework with the behavioral precision of microteaching. This randomized controlled trial aims to test the effectiveness of this integrated model in improving the clinical competencies, critical thinking, and learning satisfaction of general practice residents in a standardized training setting, compared to traditional LBL. We hypothesize that the dual-track approach will lead to superior outcomes by fostering both the “thinking” and “doing” skills essential to competent, reflective general practice.

## 2 Materials and methods

### 2.1 Study design and setting

**Figure 1:**
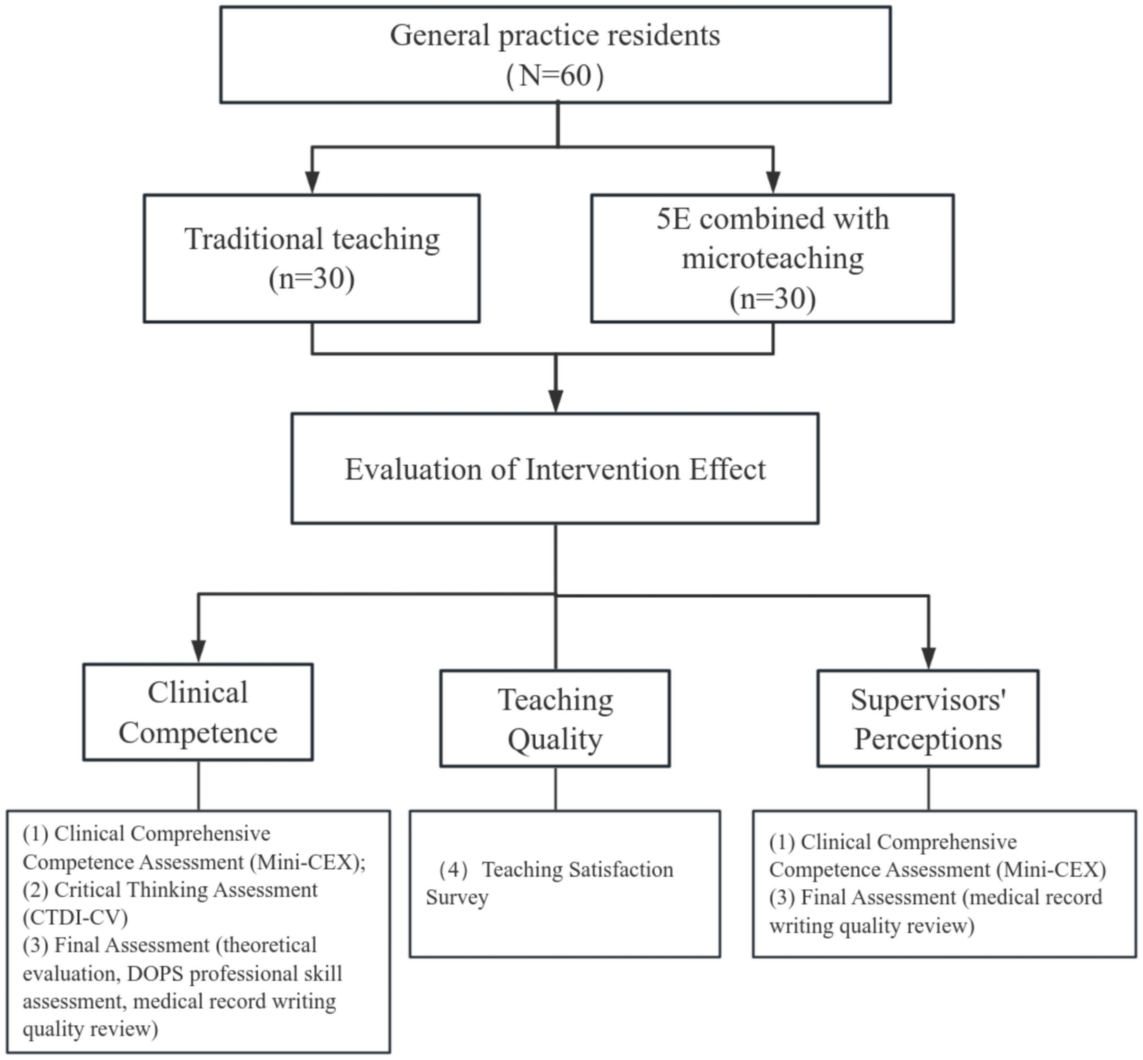
Schematic representation of the study design.

We conducted a single-center, parallel-group randomized controlled trial (RCT) at the Affiliated Hospital of Yan’an University between September 2023 and September 2025. We enrolled 60 residents undertaking standardized general practice residency training as study participants. We stratified them *a priori* by training year, age, and sex to ensure intergroup baseline comparability and mitigate potential selection bias. The training curricula and rotation schedules implemented in this study adhere strictly to the Standardized Training Content and Standards for Resident Physicians (Trial) – General Practice Training Guidelines. The Ethics Committee of the Affiliated Hospital of Yan’an University granted ethical approval for the study protocol, and we implemented all study procedures in full compliance with the hospital’s Graduate Medical Education office’s regulatory requirements. Figure 1 displays the detailed study workflow.

#### 2.1.1 Randomization and allocation concealment

Random sequence generation and group allocation were performed by an independent research assistant who was not involved in any subsequent teaching interventions or outcome assessments. A computer-generated random number table was used to allocate the 60 participants into two equal groups: the experimental group (*n* = 30) receiving the 5E-microteaching integrated model, and the control group (*n* = 30) with conventional didactic teaching. To ensure allocation concealment, the sealed, sequentially numbered opaque envelopes containing group assignment information were prepared in advance. After participants signed the informed consent form, another independent researcher opened the envelopes in enrollment order and notified participants of their grouping, with no deviations from the random sequence during the allocation process.

#### 2.1.2 Blinding strategy

Given the inherent differences in teaching intervention modalities between the two groups, blinding of participants and teaching instructors was not feasible. To minimize detection bias, a single-blinding method was strictly implemented for all outcome assessors, who were all senior clinicians not involved in the study’s teaching activities and remained unaware of participants’ group assignments throughout the assessment process. Specifically, clinical competency evaluations (including Mini-CEX and DOPS) were conducted with assessors and simulated/standardized patients blinded to group allocation; theoretical examinations and medical record quality assessments were completed in an anonymous, double-blinded manner, with all participant identifiers removed during scoring.

### 2.2 Inclusion and exclusion criteria

The inclusion criteria were as follows: (1) completion of at least 12 months of clinical rotation; (2) attendance of at least 90%; (3) general practice training for at least 1 month; and (4) signing a written consent form. The exclusion criteria were as follows: (1) suffering from acute infectious diseases or mental disorders (such as depression and anxiety) that affect regular participation in teaching activities; (2) having participated in similar teaching reform projects in the past; and (3) other force majeure factors that interfere with the implementation of the research.

### 2.3 Research methods

At the initial stage of the research, the recruitment and training of clinical teachers were completed to ensure homogeneity in the research process. Each month, we select 8 to 12 resident physicians for the general practitioner (GP) rotation. They were randomly assigned to the control and experimental groups using a random number table to ensure equal group sizes. It is necessary to ensure that the two groups remain consistent in core clinical teaching content (e.g., GP topics such as hypertension, diabetes, and chronic obstructive pulmonary disease [COPD]). The total teaching duration is 12 h (4 h of theoretical and 8 h of practical teaching), but there are significant differences in the teaching methods between the two groups.

#### 2.3.1 Control group

The traditional LBL teaching model was adopted. The teaching process was as follows:

(1) Theoretical lecture (40 min): PPT was used to explain the guidelines for chronic disease diagnosis and treatment, as well as the skill operation norms.
(2) Demonstration (20 min): The instructor showcased core skills (such as diabetic foot screening) in one go without providing detailed explanations.
(3) Student Practice (50 min): Students practice on their own, and the teacher provides walk-around help. We only answered common questions and did not provide personal microteaching feedback.
(4) Conclusion Review (10 min): The teacher reviewed the key points and did not provide structured feedback.

#### 2.3.2 Experimental group

The 5E microteaching integrated model, anchored in a dual-track cognitive-skill framework, was implemented, with differentiated learning tasks tailored to residents’ training seniority. Rather than delivering a full iterative cycle of classic microteaching, this model integrated core microteaching technical elements (video recording and video-based structured feedback) into the 5E pedagogical process, with all video recordings completed via a dedicated microteaching system for subsequent targeted evaluation. The structured teaching process was executed in five sequential stages, with the detailed implementation and duration for each stage as follows:

(1) Engagement Session (10 min): The instructor presented a realistic grassroots clinical scenario (e.g., A 68-year-old male patient with a 10-year history of hypertension, poor recent medication compliance, and concomitant fatigue) to stimulate the residents’ learning motivation. Through directed questioning, the residents were guided to propose initial clinical treatment plans for the scenario, thereby activating their pre-existing knowledge and clinical reasoning related to chronic disease management in general practice.
(2) Exploration Session (30 min): The microteaching system recorded this entire session. The residents were divided into homogeneous groups (six participants per group). Each group was assigned three structured inquiry tasks: ① reviewing evidence-based clinical practice guidelines (e.g., the Chinese Guidelines for the Prevention and Treatment of Hypertension); ② formulating individualized diagnosis and treatment plans for the presented scenario; and ③ conducting role-playing exercises to simulate the entire doctor-patient communication process for hypertension management. Peer evaluation was incorporated during group discussions, where residents mutually commented on each other’s clinical reasoning and communication strategies to facilitate collaborative learning.
(3) Explanation Session (30 min): Each group presented their formulated diagnoses and treatment plans, and role-play outcomes (8 min per group), followed by targeted, systematic explanations from the instructor. The instructor’s guidance focused on two key dimensions: ①correcting the residents’ clinical knowledge deviations and misconceptions (e.g., Long-acting calcium channel blockers are the first-line agents for elderly hypertensive patients due to their stable and sustained blood pressure control efficacy); and ② emphasizing core principles of effective doctor-patient communication (e.g., Clinicians must first identify and address patients’ concerns regarding medication side effects before discussig strategies to improve medication compliance).
(4) Elaboration Session (30 min): A progressive and complex clinical scenario was designed based on the initial case (e.g., How should the management plan be adjusted if the aforementioned patient is newly diagnosed with type 2 diabetes mellitus?). The residents were guided to transfer and apply the knowledge and skills acquired in the preceding stages to solve this complex clinical problem. Under the instructor’s direct supervision, the residents practiced standardized clinical procedural skills (e.g., diabetic foot screening). They actively compared their operational performance with international clinical protocols and benchmark teaching videos, receiving real-time verbal guidance from the instructor to immediately correct their skills.
(5) Evaluation Session (20 min): The instructor and residents jointly reviewed the video recordings from the Exploration and Explanation sessions. Combining the results of peer evaluation from the Exploitation session, the instructor objectively identified and corrected the residents’ deficiencies in clinical operations and doctor-patient communication (e.g., an Improper sequence of lung auscultation and interruptions of patients’ verbal expression during communication). Personalized feedback forms were distributed to each resident at the end of the session, clearly summarizing their individual strengths and weaknesses and providing targeted, actionable guidance for clinical skill development and knowledge enhancement.

### 2.4 Outcome indicators

To achieve the quantitative goal of the teaching intervention effect for residents, this study established a three-in-one evaluation system of “process outcome feedback”. This system combines international-standard assessment tools with subject indicators to conduct systematic evaluations of three aspects: patients’ abilities, teaching quality, and trainees’ feelings. The evaluation indicators and methods are as follows:

(1) Comprehensive clinical ability assessment: This assessment covers nine core dimensions, including medical history collection, physical examination, clinical operations, and clinical trainees’ engagement, and is scored on a nine-point scale (1–3: ability needs improvement; 4–6: meets job requirements; 7–9: outstanding ability).
(2) Critical Thinking: Critical thinking (CT) is a fundamental skill that medical students, nurses, and young physicians must possess (13). The Critical Thinking Tendency Scale - Chinese Version (CTDI-CV) was used. This scale includes assessment dimensions such as truth-seeking, openness, and cognitive maturity and is scored on a 5-point Likert scale (1 = completely inconsistent; 5 = entirely consistent) (14). The overall content validity index (CVI) of this test was 0.89, and the CVI range of each subscale tested was from 0.6 to 1. The total *α* was 0.90, and the subscale α ranged from 0.54 to 0.77 (15).
(3) Final assessment: Three modules were set according to the Examination Syllabus for the Completion of Standardized Residency Training. We conducted a theoretical evaluation by selecting questions from the standardized question bank of the National Medical Examination Center, using a computer-based test format with 80 single-choice questions and 4 case analysis questions, for a total of 100 points. We assessed professional skills using the Direct Observation of Procedural Skills (DOPS) scale (4, 16), which focuses on eight core operational competencies and uses a 9-point scoring system. The departmental quality control group, consisting of one chief physician and two attending physicians, evaluated the quality of medical record writing in a double-masked fashion. A 100-point scoring system was adopted, with assessments across five dimensions, including completeness and logicality.
(4) Teaching Satisfaction Survey: A structured questionnaire was designed based on the satisfaction evaluation framework of the Liaison Committee on Medical Education (LCME) of the United States, covering five dimensions, including teaching content and faculty level, with a total of 25 items. We used a five-point scale for scoring (1 = very dissatisfied, 5 = very satisfied (17). Three medical education experts revised and improved the questionnaire, and we assessed content validity index (CVI) and Cronbach’s *α* in a pre-survey to ensure the measurement tool’s scientific validity.

### 2.5 Sample size calculation and statistical analysis

#### 2.5.1 Sample size estimation

The sample size was *a priori* determined via power analysis based on data from a pilot study, with the Mini-Clinical Evaluation Exercise (Mini-CEX) total score as the primary outcome measure. A large effect size (Cohen’s d = 0.8) was hypothesized for the 5E-Microteaching (integrated model) relative to conventional teaching, consistent with effect sizes reported in similar medical education intervention studies. With a two-tailed significance level (*α*) set at 0.05 and a statistical power (1 − *β*) of 0.8, a two-independent-samples t-test calculation using G Power 3.1 software indicated a minimum sample size of 26 participants per group. To account for potential attrition during the study period, the final sample size was set at 30 participants per group, resulting in a total of 60 general practice residents enrolled in the trial.

#### 2.5.2 Statistical analysis

All statistical analyses were performed using R software (Version 4.3.0). Continuous variables were tested for normality using the Shapiro–Wilk test. Data following a normal distribution were expressed as mean ± standard deviation (SD) and compared between groups using the two-sample t-test. Non-normally distributed variables were reported as medians with interquartile ranges [M(Q₁, Q₃)] and analyzed using the Mann–Whitney U test. Categorical variables were summarized as frequencies (percentages) and compared using Pearson’s chi-square test, with Fisher’s exact test used when the expected frequency was less than 5. A two-tailed *p* value < 0.05 was considered statistically significant for all analyses.

## 3 Results

### 3.1 Baseline characteristics

As shown in Table 1, 60 participants were assigned to the control and experimental groups (30 each). There were no statistically significant differences between the two groups in terms of sex, age, educational background, or grade (*p* > 0.05).

**Table 1:** Basic characteristics of all participants.

Item	Control group	Experimental group	*χ*^2^/*Z*	*P*-value
Sex [*n* (%)]			*χ*^2^ = 0.07	0.787
Male	19 (63.33)	20 (66.67)		
Female	11 (36.67)	10 (33.33)		
Age (in years)	24.00 (23.00, 24.00)	24.00 (23.00, 24.00)	*Z* = −0.33	0.745
Educational background [*n* (%)]			*χ*^2^ = 0.00	1.000
Bachelor	20 (66.67)	20 (66.67)		
master	10 (33.33)	10 (33.33)		
Grade [*n* (%)]				
1st year	17 (56.67)	17 (56.67)	*χ*^2^ = 0.00	1.000
2st year	13 (43.33)	13 (43.33)		

### 3.2 Evaluation of comprehensive clinical competence

After the intervention, the Mini-CEX scores in the experimental group were significantly higher than those in the control group (*p* < 0.05). The scores were as follows: history taking (7.68 ± 0.78 vs. 8.28 ± 0.67), physical examination (7.08 ± 0.73 vs. 7.48 ± 0.69), professional quality (6.50 [6.50, 7.00] vs. 7.50 [7.50, 8.00]), clinical judgment (7.50 [7.00, 7.50] vs. 8.00 [8.00, 0.38]),doctor-patient communication (7.00 [6.50, 7.00] vs. 7.00 [7.00, 7.50]), organizational efficiency (6.50 [6.50, 7.00] vs. 7.50 [7.50, 8.00]), and comprehensive ability (7.00 [6.00, 7.50] vs. 8.00 [7.50, 8.00])(Table 2).

**Table 2:** Comparison of various Mini-CEX scores of resident physicians in the two groups.

Item	Control group	Experimental group	*t*/*Z*	*p*-value
	(*n* = 30)	(*n* = 30)		
Medical interviewing skills	7.68 ± 0.78	8.28 ± 0.67	*t* = −3.20	0.002
Physical examination skills	7.08 ± 0.73	7.48 ± 0.69	*t* = −2.18	0.033
Humanistic qualities	6.50 (6.50, 7.00)	7.50 (7.50, 8.00)	*Z* = −4.41	<0.001
Clinical judgment	7.50 (7.00, 7.50)	8.00 (8.00, 8.38)	*Z* = −4.35	<0.001
Counseling skills	7.00 (6.50, 7.00)	7.00 (7.00, 7.50)	*Z* = −2.26	0.024
Organizational/efficiency	6.50 (6.50, 7.00)	7.50 (7.50, 8.00)	*Z* = −4.54	<0.001
Evaluation of clinical comprehensive abilities	7.00 (6.00, 7.50)	8.00 (7.50, 8.00)	*Z* = −4.71	<0.001

### 3.3 CTDI-CV scores between the two groups

Group in the realistic tendency (3.00 [(3.00, 3.50] vs4.50 [4.00, 4.50]), open mind (3.50 [3.50, 4.50] vs4.50 [4.00, 4.50]), analysis ((3.50 [3.50, 4.00] vs4.00 [4.00, 4.00]) and systemic ((4.00 [3.62, 4.00] vs4.00 [4.00, 4.50]), critical thinking, self-confidence ((3.50 [3.50, 4.00] vs4.00 [4.00, 4.50] (; In terms of 3.50[3.50, 3.50] vs. 4.00[3.62, 4.00]) and the score of thirst for knowledge (25.50[25.00, 26.00] vs. 28.75[28.00, 29.00]), it showed a significant increasing trend compared with the control group (*p* < 0.05). However, there was no statistically significant difference in the cognitive maturity scores between the two groups (3.50[3.50, 4.00] vs. 4.00 [3.50, 4.00]) (*p* = 0.126) (Table 3).

**Table 3:** Comparison of CTDI-CV scores between residents in the two groups.

Item	Control group (*n* = 30)	Experimental group (*n* = 30)	*Z*	*p*-value
Seek truth	3.00 (3.00, 3.50)	4.50 (4.00, 4.50)	*Z* = −6.09	<0.001
Open mind	3.50 (3.50, 4.50)	4.50 (4.00, 4.50)	*Z* = −3.32	<0.001
Analytical ability	3.50 (3.50, 4.00)	4.00 (4.00, 4.00)	*Z* = −4.83	<0.001
Systematic ability	4.00 (3.62, 4.00)	4.00 (4.00, 4.50)	*Z* = −2.08	0.037
Self-confidence of critical thinking	3.50 (3.50, 4.00)	4.00 (4.00, 4.50)	*Z* = −3.93	<0.001
Intellectual curiosity	3.50 (3.50, 3.50)	4.00 (3.62, 4.00)	*Z* = −5.83	<0.001
Cognitive maturity	3.50 (3.50, 4.00)	4.00 (3.50, 4.00)	*Z* = −1.53	0.126
Total scores	25.50 (25.00, 26.00)	28.75 (28.00, 29.00)	*Z* = −6.48	<0.001

### 3.4 Comparison of the final assessment scores between the two groups

The average theoretical assessment score of the experimental group was higher (91.75 ± 2.15 vs. 87.57 ± 2.41, *p* < 0.001). The score for medical record writing quality in the experimental group was also higher (92.57 ± 1.92vs90.12 ± 1.86, p < 0.001). The quality score of medical record documents in the experimental group was higher (7.50[7.50, 8.50] vs. 7.50 [6.50, 7.50]; *p* < 0.001) (Table 4).

**Table 4:** Comparison of completion assessment scores between residents in the two groups.

Item	Control group (*n* = 30)	Experimental group (*n* = 30)	*T*/*Z*	*p*-value
Theoretical assessment score	87.57 ± 2.41	91.75 ± 2.15	*t* = −7.10	<0.001
Operational assessment scores	7.50 (6.50, 7.50)	7.50 (7.50, 8.50)	*Z* = −3.64	<0.001
Quality of medical record documentation	90.12 ± 1.86	92.57 ± 1.92	*t* = −5.03	<0.001

### 3.5 Comparison of teaching satisfaction between the two groups

Regarding overall satisfaction, nine participants (15.00%) expressed dissatisfaction, whereas 51 participants (85.00%) expressed satisfaction. There was a significant difference in satisfaction distribution between the two groups (*χ*^2^ = 4.71; *p* = 0.030). In the control group, eight participants (26.67%) were dissatisfied, and 22 (73.33%) were satisfied. In the experimental group, one person (3.33%) was dissatisfied, and 29 (96.67%) were satisfied (Table 5).

**Table 5:** Comparison of teaching satisfaction between residents in the two groups.

Item	Control group	Experimental group	*p*-value
Satisfaction, *n* (%)			0.03
Very satisfied	13 (43.33)	20 (66.67)	
Satisfied	9 (30.00)	9 (30.00)	
Dissatisfied	8 (26.67)	1 (3.33)	
Overall satisfaction rate, *n* (%)	22 (73.33)	29 (96.67)	0.03

## 4 Discussion

This randomized controlled trial provides robust evidence that the 5E-microteaching integrated model, anchored in a cognitive-behavioral dual-track framework, significantly enhances general practice residents’ clinical competence and critical thinking disposition compared to traditional didactic teaching. By validating this hybrid approach, the study offers a practical strategy for a challenging aspect of competency-based medical education (CBME): effectively synchronizing cognitive development with technical skill acquisition in postgraduate training. Our findings confirm that this framework offers a superior pedagogical strategy for cultivating GPs who can navigate the complexities of primary care.

The efficacy of the integrated model stems from its closed-loop synergy between theoretical reasoning and practical refinement. The 5E instructional framework (Engagement, Exploration, Explanation, Elaboration, Evaluation) guides residents through a structured clinical reasoning pathway (10). Crucially, microteaching serves as an objective feedback mechanism embedded within this process. Specifically, video recording during the “Exploration” and “Explanation” phases allows residents to review their performance externally, transforming subjective self-perception into objective self-reflection. During the “Elaboration” phase, comparing performance videos against standardized protocols facilitates the iterative correction of technical errors. This mechanism effectively bridges the “theory-practice gap” often reported in traditional apprenticeship models, where passive observation fails to translate into active competence (4, 17).

A key contribution of this study is the quantitative validation of critical thinking improvement, a competency often underexplored in prior GP training research (18). The significant gains in CTDI-CV subscales—specifically “Truth-seeking,” “Analytical ability,” and “Inquisitiveness”—suggest that the model successfully activates residents’ active learning engagement (14, 19). However, the absence of a significant change in “Cognitive Maturity” highlights a nuanced challenge. Cognitive maturity, reflecting tolerance for ambiguity and balanced judgment, likely requires longitudinal exposure to complex, multimorbid clinical realities rather than short-term training modules (20). Furthermore, this null finding may reflect the limited sensitivity of self-report instruments to detect subtle shifts in higher-order cognitive domains over brief periods. Future research should employ objective behavioral assessments, such as Situational Judgment Tests (SJTs) or Dynamic Decision-making Assessments (DDAs), to more accurately capture these deeper cognitive shifts.

From a methodological perspective, our integrated model demonstrates distinct advantages over existing educational frameworks. Unlike the One-Minute Preceptor (OMP) or Flipped Classroom (FC), which rely heavily on instructor verbal feedback or learner self-regulation (17), the 5E-microteaching model leverages technology to provide immediate, visual, and reproducible feedback. This external corrective mechanism appears more effective for skill internalization than unstructured self-reflection alone. While sharing similarities with Practice-Based Learning and Improvement (PBLI) (21), the structured five-stage design of the 5E model offers a more intuitive and operationalizable implementation pathway for residency programs.

In conclusion, this study establishes the 5E-microteaching integrated model as an effective, evidence-based pedagogy for general practice residency training. It offers a replicable framework that not only improves technical proficiency but also fosters the critical thinking essential for primary care gatekeepers. As medical education evolves towards competency-based models, integrating structured cognitive frameworks with technologically enhanced feedback loops represents a promising direction for future training reform. Specifically, integrating emerging educational technologies, such as artificial intelligence and augmented reality, holds significant potential to enhance practical skills and further learning within this model. Recent evidence suggests that these technologies effectively improve skill acquisition and retention, offering a promising avenue for the digital expansion of the 5E-microteaching framework (22).

### 4.1 Limitations and future directions

Despite the rigorous randomized controlled trial (RCT) design, this study has several limitations. First, no baseline measurements were taken for the primary outcomes—clinical competence (Mini-CEX) and critical thinking (CTDI-CV). Although randomization ensured between-group comparability at baseline, the absence of pre-intervention data precluded within-group pre-post comparisons, thereby limiting the internal validity of the intervention effects. Second, the single-center design with a small sample size (*n* = 60) may limit generalizability. All participants were from a single tertiary hospital with similar training resources; thus, the findings may not apply to residents in primary care or in other institutions with differing educational contexts. Third, the intervention was implemented during a single rotation with only immediate post-intervention assessments. The lack of long-term follow-up prevents evaluation of the sustainability of improvements in clinical skills and critical thinking, as well as their translation into real-world clinical outcomes (e.g., diagnostic accuracy, rational prescribing, patient satisfaction). Fourth, the study did not include separate control groups receiving the 5E model or microteaching alone, which precludes identification of the unique contribution of each component to the observed synergistic effect.

Additionally, a formal re-teaching and re-feedback cycle was not incorporated, further limiting methodological robustness. Fifth, participants were aware of their involvement in an educational study, which may have introduced a Hawthorne effect—whereby individuals perform better due to increased attention—although randomization may partially mitigate this bias. Sixth, despite this blinding, complete blinding was challenging for behavioral assessments (e.g., communication skills), potentially introducing detection bias.

To address the above limitations and further advance research on the integrated 5E-Microteaching (integrated model)in general practice residency training, five targeted directions are proposed. First, subsequent studies should incorporate comprehensive baseline assessments of all core outcome indicators—including clinical competence (Mini-CEX), critical thinking disposition (CTDI-CV), and theoretical and procedural skills—to enable both intra-group pre-post comparisons and inter-group comparisons of change scores. This will more accurately quantify the intervention’s effect size and strengthen internal validity. Second, a multicenter randomized controlled trial with a larger and more diverse sample is warranted. Recruiting general practice residents across different regions would allow verification of the model’s adaptability and generalizability, providing robust evidence to support broader implementation. Third, long-term follow-up studies should be designed with extended intervention and assessment periods. Implementing the intervention throughout the entire standardized training cycle, with follow-up evaluations at 6 months, 1 year, and 2 years post-intervention, would enable assessment of the long-term retention of clinical skills and critical thinking, as well as the model’s impact on real-world clinical outcomes—thereby clarifying its sustained educational value. Fourth, future research should include separate intervention groups receiving the 5E model or the microteaching-based feedback approach alone, alongside traditional teaching and integrated model groups. A four-group comparative design would allow quantitative analysis of the unique contributions of each component and the synergistic effects of their integration, facilitating further optimization of the model to enhance teaching efficiency and targeting. Fifth, the integrated model could be further enhanced by incorporating cutting-edge digital educational tools. AI-driven video analysis may be employed to provide personalized skill feedback within the microteaching framework. At the same time, virtual reality (VR)-based clinical scenarios could be integrated into the engagement and inquiry stages to create immersive primary care contexts. Such technological enhancements would improve the scalability and precision of the cognitive-behavioral dual-track training framework, ultimately contributing to a standardized and scalable “5E-Microteaching (integrated model) protocol for competency-based general practice education reform.

## 5 Conclusion

This study demonstrates that integrating the 5E teaching model with video-based feedback grounded in microteaching principles effectively enhances clinical competence, critical thinking disposition, and educational satisfaction among general practice residents. The proposed model forms a coherent learning cycle—engagement, exploration, explanation, elaboration, and evaluation—within which video-based feedback facilitates the deep integration of learning with practice and the alignment of reasoning with clinical action. These findings offer a practical and competency-oriented reference for advancing general practice education.

## Data Availability

The raw data supporting the conclusions of this article will be made available by the authors, without undue reservation.
